# Henry Howard Keith, D.D.S.

**Published:** 1899-05

**Authors:** 


### Obituary.

### HENRY HOWARD KEITH, D.D.S.

Henry Howard Keith, the only son of Amos B. and Katie M. Keith, was born at Salem, Mass., June 14, 1847. He resided in Boston until the beginning of the Civil War, when the family moved to Chicago. After working a short time in a machine-shop to learn the use of tools he was apprenticed to his uncle, who was a jeweller. In 1864 Dr. Keith went to Philadelphia and entered the laboratory of Dr. Charles Essig. He made such rapid progress that when Dr. Essig moved his laboratory to Baltimore, in 1868, he took Dr. Keith with him, and while there he met the lady who afterwards became his wife. After spending about two years in Baltimore he went to Newark, N. J., where he worked for Drs. DaCamare and Pinney until he was married, in January, 1871. His wife was Miss Nina L. Benteen, of Baltimore.

He came to St. Louis in February, 1871, and in the fall of the same year opened a laboratory. He was successively with Drs. Morrison, Eames, Park, McKellops, and Lange. He attended the Missouri Dental College, and graduated in 1873, and afterwards held positions in the same college, in 1875–76, as demonstrator of Mechanical Dentistry, and was professor of Mechanical Dentistry from 1876 to 1879 inclusive.

Dr. Keith's talents lay in the direction of plate work, in which he had no superior, though he had a large practice in operative work. His heart was in his profession, and he spared neither time nor pains when engaged in a difficult piece of work; and the greater the difficulties the more he enjoyed overcoming them,—in which he seldom failed. As an operator he was equally skilled, and was extremely gentle and considerate of his patients.

His chief characteristic seemed to be a desire to help the younger members of the profession. Every one coming to St. Louis met with a pleasant welcome from him, and he always did what he could to advance them.

At Lake Minnetonka, in 1888, he contracted the disease that finally caused his death. His summers, for seven years past, have been spent in Asheville, N. C. He returned to St. Louis in September, 1898, and though in very poor health, he resumed his practice. His health failed very fast, and his death was the result of peritonitis. He died January 26, 1899, and was cremated at the St. Louis Crematory, as he had desired. His ashes will be buried at Riverside Cemetery at Asheville.

As an instructor he was one of the few men who seemed capable of imparting his knowledge so that the one instructed could not fail to grasp the ideas of the master mind. He was a member of a sketch club and well versed in photography. For that reason he was an adept in illustrating his subject. His office was a study in itself for neatness, convenience, and all that was new in modern dentistry. His laboratory was equal to that of his office. He was considered one of the best continuous-gum workers in this country. All his work in this particular line had the finish of a master artist.

He was a man who was continually striving to accomplish something that would advance his profession. He was an active member of both State and city societies. Of the latter he served four years as recording secretary, and in 1882 was the president, and no better drawing card could be announced than the mere statement that Dr. Keith would either read a paper or give a talk on some dental subject. As a professional man he was a model. He was never known to speak ill of any professional brother so as to advance himself in the estimation of his patients. His professional liberality was one of his many good traits that should be observed by us all. He was always willing to assist any dentist, both financially and professionally, and nothing would give him greater pleasure than to impart his practical knowledge to any brother needing his advice. He was a frequent contributor to the dental journals.

John G. Harper,
Walter M. Bartlett,
Joseph G. Pfaff,
*Committee.*

St. Louis Dental Society.

